# Use of a linearization approximation facilitating stochastic model building

**DOI:** 10.1007/s10928-014-9353-5

**Published:** 2014-03-13

**Authors:** Elin M. Svensson, Mats O. Karlsson

**Affiliations:** Department of Pharmaceutical Biosciences, Uppsala University, P.O. Box 591, 751 24 Uppsala, Sweden

**Keywords:** Linearization, Random effects, Nonlinear mixed effects models, Pharmacometrics, Diagnostics

## Abstract

The objective of this work was to facilitate the development of nonlinear mixed effects models by establishing a diagnostic method for evaluation of stochastic model components. The random effects investigated were between subject, between occasion and residual variability. The method was based on a first-order conditional estimates linear approximation and evaluated on three real datasets with previously developed population pharmacokinetic models. The results were assessed based on the agreement in difference in objective function value between a basic model and extended models for the standard nonlinear and linearized approach respectively. The linearization was found to accurately identify significant extensions of the model’s stochastic components with notably decreased runtimes as compared to the standard nonlinear analysis. The observed gain in runtimes varied between four to more than 50-fold and the largest gains were seen for models with originally long runtimes. This method may be especially useful as a screening tool to detect correlations between random effects since it substantially quickens the estimation of large variance–covariance blocks. To expedite the application of this diagnostic tool, the linearization procedure has been automated and implemented in the software package PsN.

**Electronic supplementary material:**

The online version of this article (doi:10.1007/s10928-014-9353-5) contains supplementary material, which is available to authorized users.

## Introduction

Population pharmacokinetics (PK) and pharmacodynamics (PD) models, i.e. pharmacometrics, play an increasingly important role in pharmaceutical sciences and drug development [1]. Nonlinear mixed effects (NLME) models are frequently used to describe population PK/PD and are composed of a structural component (fixed effects) and a stochastic component (random effects). Fixed effects describe the typical parameter values in a population and the effects of covariates. Random effects handle unexplained variability and can be further divided into variability assigned to parameters and residual variability (RV) assigned to the observations. Parameters can vary on multiple levels: between subjects (BSV), between occasions (BOV), between studies, etc.

For NLME, especially when used in simulations, the stochastic components of the model are crucial but the development procedure can be laborious. Despite a vast increase in available computational power during the past decade, the time for parameter estimation can still be a limiting factor since the complexity of the used models and the amount of fitted data typically are increasing likewise. For highly nonlinear models numerical instability of parameter estimation with common gradient based, local methods may cause further problems. Individual parameter estimates, the empirical Bayes estimates (EBEs), can be used as a diagnostic tool to aid the development procedure. However, this practice has notable shortcomings when data are uninformative on the individual level and shrinkage towards the population mean occurs. Shrinkage can cause diagnostics based on EBEs to mask, falsely induce or distort the shape of random effects relationships [2, 3]. The objective of this work was to develop and assess a fast and stable method which is not sensitive to shrinkage for diagnostics of BSV, BOV and RV model components. The here proposed method, henceforth called ‘linearization’, is based on a first-order conditional estimation (FOCE) linear approximation which has previously successfully been applied for testing of covariates [4]. The linearization was evaluated in the modeling software NONMEM with a variety of different models and datasets. The results are presented as comparisons between the linearization and the corresponding nonlinear model, both in terms of estimation performance and runtimes.

## Methods

### Population models and linearization

NLME models commonly used in population PK/PD can be formally represented by

1 $$ y_{ij} = f(\overrightarrow {p}_{i} ,\overrightarrow {x}_{ij} ) + h_{ij} $$

where *y*_*ij*_ is the data point of the *i*th individual’s *j*th observation, *f* is a model that relates the vector of individual parameters $\vec{p}_{i}$ and the vector of independent variables $\vec{x}_{ij}$ (for example dose and time) to the observations and $h_{ij}$is a model for the residual error. The individual parameters can be described as

2 $$ \overrightarrow {p}_{i} = \overrightarrow {p} \left( {\overrightarrow {\theta } ,\;\overrightarrow {\eta }_{i} ,\;\overrightarrow {g} \left( {\overrightarrow {z}_{i} ,\;\overrightarrow {\theta }_{{\overrightarrow {g} }} } \right)} \right) $$

where $\vec{p}$ is the vector of models relating the typical parameter values in the population $\vec{\theta }$, the parameter-specific variability $\vec{\eta }_{i}$ and the vector of covariate functions $\vec{g}$, including covariate observations $\vec{z}_{i}$ and typical population values for respectively covariate-parameter relation $\vec{\theta }_{{\vec{g}}}$. The parameter-specific variability can have multiple levels simultaneously and is usually assumed to be normally distributed with mean 0 and variance–covariance matrix Ω. BSV and BOV are frequently modeled by assuming an exponential relationship to obtain log-normal parameter distributions. Under this assumption, parameter *k* in individual *i* with *L* levels of parameter-specific variability can be describe by the following model

3 $$ p_{ki} = \theta_{k} \times g_{k} \left( {\overrightarrow {z}_{i} ,\;\overrightarrow {\theta }_{{\overrightarrow {g} }} } \right) \times e^{{\left( {\sum\limits_{l = 1}^{L} {\eta_{lki} } } \right)}} $$

The residual error model is commonly modeled as a function of $\vec{\varepsilon }_{ij}$ which is assumed to be normally distributed with mean 0 and variance–covariance matrix Σ. Common forms of *h*_*ij*_ are additive error (*h*_*ij*_ = *ɛ*_*ij*_), proportional error $(h_{ij} = f\left( {\vec{p}_{i} ,\vec{x}_{ij}  } \right) \times \varepsilon_{ij} )$ or a combined error with both an additive and a proportional element $(h_{ij} = f\left( {\vec{p}_{i} ,\vec{x}_{ij}  } \right) \times \varepsilon_{1ij} + \varepsilon_{2ij} )$. Examples of extensions and more flexible forms have been described in the following references [5–8].

Model parameters can be obtained by maximum likelihood estimation where the estimated parameter values maximizes the likelihood of the observed data [9]. Maximizing the likelihood is equivalent to minimizing the twice negative log-likelihood (objective function value, OFV). Evaluation of the function for the log-likelihood of NLME models require integration steps that are computationally demanding and a simpler linear approximation of the model can be evaluated instead. We here assess the possibility of utilizing partial derivatives and EBEs from evaluation of a nonlinear base model to assess the improvement in model fit by additional random effects through estimation of extensions implemented on the linear approximation of the base model. The linear approximation consists of first-order Taylor expansions initially around $\vec{\varepsilon }_{ij} = \vec{0}$ (Eq. 4) and subsequently around $\vec{\eta }_{i} = \vec{\hat{\eta }}$ where $\vec{\hat{\eta }}_{i}$ represents the EBE’s of $\vec{\eta }_{i}$ (Eq. 5, the linearized model).

4 $$ y_{ij} \approx f\left( {\overrightarrow {p}_{i} ,\;\overrightarrow {x}_{ij} } \right) + \left. {\sum\limits_{v = 1}^{\tau } {\frac{{\partial h_{ij} }}{{\partial \varepsilon_{ijv} }}} } \right|_{{\overrightarrow {\varepsilon }_{ij} = \overrightarrow {0} }} \varepsilon_{ijv}^{*} $$

5 $$ y_{ij} \approx \left. {f\left( {\vec{p}_{i} ,\vec{x}_{ij}  } \right)} \right|_{{Q_{0} }} + \left. {\mathop \sum \limits_{l = 1}^{m} \frac{\partial f}{{\partial \eta_{li} }}} \right|_{{Q_{0} }} \left( {\eta_{li}^{*} - \hat{\eta }_{li} } \right) + \left. {\mathop \sum \limits_{v = 1}^{\tau } \frac{{\partial h_{ij} }}{{\partial \varepsilon_{ijv} }}} \right|_{{Q_{0} }} \varepsilon_{ijv}^{*} + \left. {\mathop \sum \limits_{v = 1}^{\tau }  \mathop \sum \limits_{l = 1}^{m} \frac{\partial }{{\partial \eta_{li} }}\left( {\left. {\frac{{\partial h_{ij} }}{{\partial \varepsilon_{ijv} }}} \right|_{{Q_{0} }} } \right)} \right|_{{Q_{0} }} \varepsilon_{ijv}^{*} \left( {\eta_{li}^{*} - \hat{\eta }_{li} } \right) $$

where ${\text{Q}}_{0} = \, (\vec{\varepsilon }_{ij} = \vec{0},\,\vec{\eta }_{i} = \vec{\hat{\eta }}_{i} )$, m is the number of element in $\vec{\eta }_{i}$ and τ is the number of elements in $\vec{\varepsilon }_{ij} .$ The partial derivatives and EBE’s are obtained by evaluating the nonlinear base model with the first order conditional estimation method with interaction (FOCE-I) in NONMEM (see section Software). If the error model is simply additive FOCE without interaction can be used. The FOCE and FOCE-I methods also utilize Taylor expansions and are described in NONMEM users guide-part VII [10]. The parameters marked with asterisks in Eqs. 4 and 5 are the unknown parameters of the linearized model that remain to be estimated. A comprehensive example code is provided as supplementary material. Since the iterative calculation of fixed effects parameters and partial derivatives are time consuming steps, especially for models including large variance–covariance matrixes, the estimation of a beforehand linearized extended model (where iterative calculations only are needed for the random effects) is magnitudes faster than estimation of the nonlinear extended model.

### Software

NONMEM versions 7.2 and 7.3 beta (ICON Development Solutions, Hanover, MD, USA) [10] with the estimation method FOCE-I were used for the analysis. To overcome sensitivity to local minima when estimating individual parameters (*η*-values) the new NONMEM option MCETA was utilized. The default initial value for all *η* is zero. MCETA allows the user to define a number of vectors containing random samples of initial *η*-values that should be tested in addition to zero, whichever supplies the lowest OFV will be used as initial value in the estimation. The numbers in each vector of initial *η*-values are randomly drawn from the normal distribution with mean zero and variance–covariance matrix Ω. With a large enough number of initial *η*-values tested, the probability of the estimation to end up in a local minimum can be decreased.

Models were executed through PsN, using Piranha for documentation and creation of run records [11–13].

### Evaluated model extensions

**Fig. 1:**
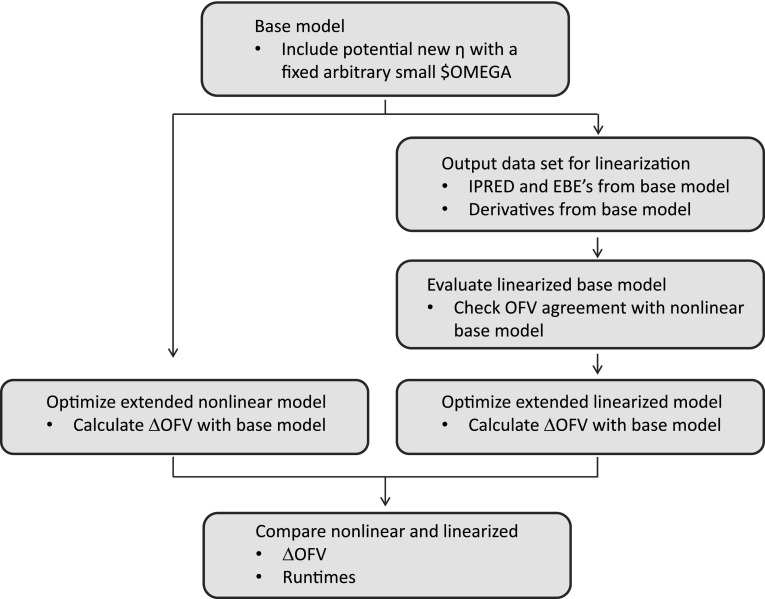
Work flow to compare performance of nonlinear and linearized models in NONMEM

A comprehensive overview of the work flow for comparing standard nonlinear and linearized models is given in Fig. 1. The evaluated RV models were extensions to an additive, a proportional or a combined base error model. The evaluated extensions were:

- BSV of the residual error [14]
- A power relation with the individual model predictions (F), implemented as $h_{ij} = h_{ij(base)} \times F^{\theta }$
- Autocorrelation, i.e. serial correlation between the error of observations consecutive in time [5]
- Time dependent residual error, implemented as a step function [5]

The NONMEM code of the RV extensions is provided in the supplementary material. For extensions of the BOV and/or the correlation structure, a base model already including some BSV terms was used while for extensions of the BSV structure the base model only included RV. To this model additional parameter-specific variability were added and/or the structure of the variance–covariance block changed. To enable estimation of the linearized model with additional variability parameters, the partial derivatives of the model with respect to the new parameters must be known. The partial derivatives can be obtained in NONMEM by including the code for the additional variability parameters already in the nonlinear base model but fixed to an arbitrary small value (fixing it to zero would result in that no derivatives are calculated).

All extended models were estimated with FOCE-I both in the linearized form and as standard nonlinear models. The results were evaluated by comparing the difference in OFV (ΔOFV) between the extended model and the base model for the two approaches respectively. The runtimes for the estimation step were extracted from the result files. The runtime comparisons should be interpreted in terms of magnitude and not as exact figures, since all estimations were carried out at a cluster with many nodes, which have somewhat different capacities and are randomly assigned.

### Datasets

Three real data examples with previously developed models with diverse residual error structures were used to illustrate the methodology.

1. Moxonidine [14]: The data were obtained from a phase II multicenter study of oral moxonidine in patients with congestive heart failure and contained 1,022 observations from 74 patients. The structural model describing the data was a one-compartment model with first-order absorption and an additive residual error model.
2. Pefloxacin [15, 16]: The data were obtained from critically ill patients receiving 1 h intravenous infusions of pefloxacin and contained 337 observations from 74 patients. The structural model was a one-compartment model and a proportional residual error model.
3. Ethambutol [17]: The data were obtained from two prospective studies in tuberculosis patients receiving a standard treatment regimen including ethambutol. A total of 1869 observations from 189 patients were included in the dataset. The structural model was a one-compartment model with first-order absorption through one transit compartment and a combined residual error model.

## Results

**Fig. 2:**
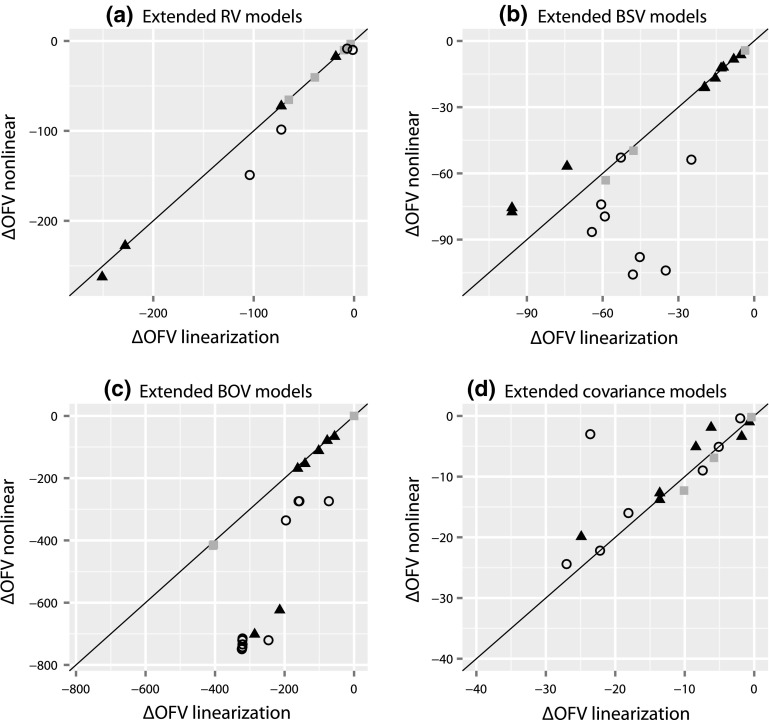
Difference in OFV between base and various extended RV (**a**), IIV (**b**), IOV (**c**) and covariance (**d**) models (described in methods), after estimation with the nonlinear vs. the linearized approach for the moxonidine (*black triangles*), pefloxacin (*grey squares*) and ethambutol (*open circles*) data examples

The agreement between the ∆OFV of the nonlinear and the linearized extended RV models were found to be good (Fig. 2a). Also for extended correlations structures the agreement was good (Fig. 2d). For extended BSV and BOV models the agreement was acceptable (Fig. 2b, c). The deviations between the nonlinear and linearized model were the largest for instances where the value of one or several fixed effect parameter(s) in the nonlinear model were notably changed by the extension. The extended linearized models do not allow a change of the fixed effects since those values are incorporated in the predictions and derivatives obtained from the base model. The linearized analysis identified the same extended models to be significant improvements as the conventional nonlinear analysis in all cases except in four, resulting in an accuracy of 96 % (88 of 92). In the four deviating cases the ΔOFVs were very close to the significance level (following Chi square distribution, α = 0.05).

The total runtimes for the linearized models using the three example datasets were markedly shorter compared to the corresponding nonlinear models (Table 1). When the linearization of the base model is successful, the OFV value of the linearized version and that of the corresponding nonlinear model estimated with FOCE-I should be very similar. This was the case for all evaluated models with an additive residual error model. However, for models with proportional or combined residual error models, a number of cases were discovered where the OFV values differed greatly and a lower OFV value was obtained for the nonlinear base model. Closer evaluation of the results revealed that this deviation between OFV values was caused by certain subjects for whom the individual contributions to the OFV (iOFV) were notably higher, whereas for the majority of subjects the iOFV values of the two methods matched well. For the deviating subjects the estimation of individual *η*-values had failed in the linearized model due to issues with local minima. Transformation to render the error additive in the transformed space or utilization of the MCETA option resolved the problem for all evaluated cases.

**Table 1:** Comparison of total runtimes for nonlinear and linearized models using the three example datasets

Data	Total runtime nonlinear (s)	Total runtime linearized (s)	Fraction time required linearized (%)
Moxonidine	735.1	106.5	14.5
Pefloxacin	47.78	6.96	14.6
Ethambutol	103082	25983^a^	25.2

^a^Executed with MCETA = 1,000

## Discussion

A novel diagnostic method for evaluation of random effects was successfully developed and evaluated. The linearization was found to accurately identify significant extensions of models’ stochastic components with notably decreased runtimes as compared to standard nonlinear analysis. The three examples used had relatively short runtimes also in their nonlinear form. When the linearization was applied to more complex models, an even more substantial decrease in runtimes (>50×) was observed. For a PD-model describing the effect of docetaxel on neutrophil counts (extension of [18] ) utilizing the full random effects approach (FREM [19] ) the runtime was decreased from 3 h and 50 to 5 min (2.2 %). For a PK model of bedaquiline plus two metabolites including a large covariance block (extension of [20] ) the runtime was reduced from 15 h and 52 to 6 min (0.6 %). For a PK-model of rifabutin plus metabolite using a dataset combining 14 clinical studies (unpublished) for which the runtime of the base nonlinear model was several day, the estimation of 6 additional BSV parameters and extending the variance–covariance matrix from a diagonal to a full 12 × 12 block structure took only 18 and 89 min, respectively.

When applied to models with a proportional or a combined residual error structure the linearization was found to be sensitive to local minima when assigning the individual *η*-values. This happens due to the potential shape distortion of the individual EBE likelihood profiles that can be caused by the interaction term (including the partial derivative with respect to both *ɛ* and *η* of the residual error model) in the linearized model. The interaction term will always be zero for additive residual error models which explains why the problem was never observed in this case. Care must be taken to ensure that the OFV of the linearized base model agrees with the corresponding nonlinear model. If a deviation is detected, simple work-arounds can preclude potential deviations. Transformations can render the error model additive in the transformed space, for example log-transformation for model with proportional error structure. Another solution is the MCETA option, available in NONMEM from version 7.3.0 [21], which decreases the risk of issues with local minima by supplying multiple initial estimates for the individual *η*-values. With MCETA values of between 10 and 1,000 all evaluated linearized models were able to obtain the same OFV value as the corresponding nonlinear model. However, run times were somewhat increase by use of the MCETA option. Yet another potential solution could be to input the EBE’s from the nonlinear base model as initial estimates for individual *η*-values in the linearized model. This is possible with the ETAS option, also available in NONMEM from version 7.3.0. The ETAS option should be faster than the MCETA option since it only tests one set of initial estimates instead of several, but it may be less reliable. In cases when the shape of the likelihood profiles contain multiple local minima, as have been observed for linearized models with proportional or combined error structures, the risk of terminating in local minima is substantial even with initial estimates close to the optimal values and therefore the use of the MCETA option could be safer.

To simplify the use of this novel methodology, an option called ‘linearize’ has been implemented in PsN (available from version 3.7.0). The procedure requires the provision of a nonlinear model, optimizes the parameters and outputs predictions and derivatives in a new dataset. This dataset constitutes the input for an automatically created linearized version of the same model. The linearized model can serve as starting point for evaluation of manually coded extensions of the random effects. Model building could potentially be made even more efficient by automated testing of a library of RV models, comparable to the stepwise covariate search method (the SCM) already implemented in PsN. Since the values of fixed effects are not estimated in the linearized model the method should be viewed as a diagnostic tool. It is recommended to re-estimate with standard nonlinear methods once the best extended model is identified.

In conclusion, the successful use of a linear approximation method for fast diagnosis of a broad range of extended random effects models was demonstrated. The method may be especially valuable as a screening tool to detect correlations between random effects since estimating large variance–covariance blocks often is a computationally demanding and time-consuming process but can be carried out magnitudes faster with the linearization.

## Electronic supplementary material

Below is the link to the electronic supplementary material.

Supplementary material 1 (DOCX 20 kb)
