# Supplementary material for: Use of a linearization approximation facilitating stochastic model building
Source: J Pharmacokinet Pharmacodyn. 2014 Mar 13;41(2):153–8. doi: 10.1007/s10928-014-9353-5 (PMC3969514; doi:10.1007/s10928-014-9353-5)
Supplement: Supplementary file 1 — Supplementary material 1 (DOCX 20 kb) [file 10928_2014_9353_MOESM1_ESM.docx]

## Supplementary material

### Example code

**; model example original.mod**

$PROBLEM MOXONIDINE
$INPUT ID VISI AGE SEX WT ACE DIG DIU TAD TIME CLCR AMT SS II DV
$DATA data.csv IGNORE=@
$SUBROUTINE ADVAN2 TRANS1

$PK

TVCL = THETA(1)
TVV = THETA(2)

CL = TVCL*EXP(ETA(1))
V = TVV*EXP(ETA(2))
KA = THETA(3)*EXP(ETA(3))
ALAG1 = THETA(4)
K = CL/V
S2 = V

$ERROR

IPRED = LOG(.025)
W = THETA(5)
IF(F.GT.0) IPRED = LOG(F)
IRES = IPRED-DV
IWRES = IRES/W

Y = IPRED+ERR(1)*W

$THETA (0,26.6) ; TVCL
$THETA (0,1.43) ; TVV
$THETA (0,4.45) ; TVKA
$THETA (0,.240) ; LAG
$THETA (0,.33) ; RES ERR

$OMEGA BLOCK(1) 0.0404 ; IIV CL
$OMEGA BLOCK(1) 0.0270 ; IIV V
$OMEGA BLOCK(1) 2.56 ; IIV KA
$SIGMA 1 FIX ; Estimating SIGMA instead of THETA(5) works just as well

$ESTIMATION METHOD=COND MAXEVALS=9999 ; INTERACTION not necessary, additive error

**; Nonlinear base model with table output suitable for input in the linearized model**; Created automatically by PsN command: –linearize original.mod

$PROBLEM MOXONIDINE
$ABBREVIATED COMRES=3
$INPUT ID VISI AGE SEX WT ACE DIG DIU TAD TIME CLCR AMT SS II DV
$DATA data.csv IGNORE=@
$SUBROUTINE ADVAN2 TRANS1

$PK

TVCL = THETA(1)
TVV = THETA(2)

CL = TVCL*EXP(ETA(1))
V = TVV*EXP(ETA(2))
KA = THETA(3)*EXP(ETA(3))
ALAG1 = THETA(4)
K = CL/V
S2 = V

$ERROR

IPRED = LOG(.025)
W = THETA(5)
IF(F.GT.0) IPRED = LOG(F)
IRES = IPRED-DV
IWRES = IRES/W

Y = IPRED+ERR(1)*W

"LAST
" COM(1)=HH(1,2)
" COM(2)=HH(1,3)
" COM(3)=HH(1,4)

$THETA (0,26.6) ; TVCL
$THETA (0,1.43) ; TVV
$THETA (0,4.45) ; TVKA
$THETA (0,.240) ; LAG
$THETA (0,.33) ; RES ERR

$OMEGA BLOCK(1) 0.0404 ; IIV CL
$OMEGA BLOCK(1) 0.0270 ; IIV V
$OMEGA BLOCK(1) 2.56 ; IIV KA
$SIGMA 1 FIX

$ESTIMATION METHOD=COND MAXEVALS=9999 ; INTERACTION not necessary, additive error

$TABLE ID DV MDV IPRED=OPRED VISI AGE SEX WT ACE DIG DIU TAD TIME CLCR AMT SS II H011 G011 G021 G031 ETA1 ETA2 ETA3 COM(1)=D_EPSETA1_1 COM(2)=D_EPSETA1_2 COM(3)=D_EPSETA1_3 NOPRINT NOAPPEND ONEHEADER FILE=run100_linbase.dta

**; Linearized base model**; Simultaneously created by PsN with the same command (-linearize original.mod)

$PROBLEM MOXONIDINE

$INPUT ID DV MDV OPRED VISI AGE SEX WT ACE DIG DIU TAD TIME CLCR AMT SS II D_EPS1 D_ETA1 D_ETA2 D_ETA3 OETA1 OETA2 OETA3 D_EPSETA1_1 D_EPSETA1_2 D_EPSETA1_3
$DATA run100_linbase.dta IGNORE=@

$PRED

BASE1=D_ETA1*(ETA(1)-OETA1)
BASE2=D_ETA2*(ETA(2)-OETA2)
BASE3=D_ETA3*(ETA(3)-OETA3)

BSUM1=BASE1+BASE2+BASE3
BASE_TERMS=BSUM1

IPRED=OPRED+BASE_TERMS

ERR1=EPS(1)*(D_EPS1+D_EPSETA1_1*(ETA(1)-OETA1))
ERR2=EPS(1)*(D_EPSETA1_2*(ETA(2)-OETA2))
ERR3=EPS(1)*(D_EPSETA1_3*(ETA(3)-OETA3))

ESUM1=ERR1+ERR2+ERR3

ERROR_TERMS=ESUM1

Y=IPRED+ERROR_TERMS

$OMEGA BLOCK(1) 0.0811577 ; IIV CL
$OMEGA BLOCK(1) 2.80609 ; IIV V
$OMEGA BLOCK(1) 0.0513593 ; IIV KA

$SIGMA 1

$ESTIMATION METHOD=COND INTERACTION

**; Code examples of extended RV models**; The left column shows the original codes of the base models and right column the adjusted codes of
; the extended models

**; BSV of the residual error**

;Nonlinear

W = THETA(5) 🡪 W = THETA(5)*EXP(ETA(4))

;Linearized

ERROR_TERMS=ESUM1 🡪 ERROR_TERMS=ESUM1*EXP(ETA(4))

**; Power relation with individual model predictions**

;Nonlinear

W= THETA(5) 🡪 W= THETA(5)
IF(F.GT.0) W = THETA(5)*F**THETA(7)

;Linearized

ERROR_TERMS=ESUM1 🡪 ERROR_TERMS=ESUM1
 FDV=EXP(IPRED) ; Transformation needed since
 ; IPRED is on log scale
 IF(FDV.GT.0) ERROR_TERMS=ESUM1*FDV**THETA(1)

**; Time dependent residual error**

;Nonlinear

W = THETA(5) 🡪 W = THETA(5)
 IF(TAD.GE.2) W =THETA(6)

;Linearized

ERROR_TERMS=ESUM1 🡪 ERROR_TERMS=ESUM1
 IF(TAD.GE.2) ERROR_TERMS = ESUM1*THETA(1)

**; Autocorrelation**

;Nonlinear and linearized

; Code added first in $ERROR for nonlinear and first in $PRED for linearized modes

"FIRST
" USE SIZES, ONLY: NO
" USE NMPRD_REAL, ONLY: C=>CORRL2
" REAL (KIND=DPSIZE) :: T(NO)
" INTEGER (KIND=ISIZE) :: I,J,L

"MAIN
"C If new ind, initialize loop
" IF (NEWIND.NE.2) THEN
" I=0
" L=1
" OOCC=VISI
" OID=ID
" END IF
"C Only if first in L2 set and if observation
" IF (NEWL2.EQ.1.AND.EVID.EQ.0) THEN
" I=I+1
" T(I)=TIME
" IF (OID.EQ.ID.AND.OOCC.NE.VISI) THEN
" L=I
" OOCC=VISI
" END IF
" DO J=L,I
" C(J,1)=EXP((-0.6931/THETA(6))*(T(I)-T(J))) ; THETA(1) for linearized
" ENDDO
" END IF
